# Targeted Victimization and Suicidality Among Trans People: A Web-Based Survey

**DOI:** 10.1089/lgbt.2017.0011

**Published:** 2018-04-01

**Authors:** Galit Zeluf, Cecilia Dhejne, Carolina Orre, Louise Nilunger Mannheimer, Charlotte Deogan, Jonas Höijer, Regina Winzer, Anna Ekéus Thorson

**Affiliations:** ^1^ Department of Public Health Sciences, Karolinska Institutet, Stockholm, Sweden.; ^2^ Department of Clinical Neuroscience, Centre for Psychiatry Research, Karolinska Institutet, Stockholm, Sweden.; ^3^ ANOVA, Andrology, Sexual Medicine, and Transgender Medicine, Karolinska University Hospital, Stockholm, Sweden.; ^4^ Department of Health and HIV-Prevention, the Swedish Federation for LGBTQ Rights (RFSL), Stockholm, Sweden.; ^5^ Department of Learning, Informatics, Management and Ethics, Karolinska Institutet, Stockholm, Sweden.; ^6^ Department of Communicable Disease Control and Health Protection, the Public Health Agency of Sweden, Stockholm, Sweden.; ^7^ Unit of Biostatistics, Institute of Environmental Medicine, Karolinska Institutet, Stockholm, Sweden.; ^8^ Department of Living Conditions and Lifestyles, the Public Health Agency of Sweden, Stockholm, Sweden.

**Keywords:** life satisfaction, suicide, transgender, victimization

## Abstract

***Purpose:*** The aim of this study was to investigate the associations between a series of empirically known risk and protective factors and suicidality among trans people in Sweden.

***Methods:*** Participants were self-selected anonymously to a web-based survey conducted in 2014. Univariable and multivariable logistic regression analyses were performed to assess associations between contributing factors and suicide ideation in the past 12 months and lifetime suicide attempts.

***Results:*** The analysis included 796 trans individuals, between 15 and 94 years of age, who live in Sweden. A total of 37% of respondents reported that they have seriously considered suicide during the past 12 months and 32% had ever attempted a suicide. Offensive treatment during the past three months and lifetime exposure to trans-related violence were significantly associated with suicidality. Less satisfaction with contacts with friends and acquaintances and with one's own psychological wellbeing were associated with suicide ideation in the past 12 months. Lack of practical support was associated with lifetime suicide attempts.

***Conclusions:*** Our findings show that suicidality is directly correlated with trans-related victimization. Preventing targeted victimization is, therefore, a key preventive intervention against this elevated suicidality.

## Introduction

Suicide is a major public health concern.^1^ In Sweden, suicide is the leading cause of death among men between the ages of 15–44 and the second cause of death for women in the same age group.^2^ According to national estimates, 5% of the general population in Sweden had seriously considered suicide in the past year.^3^ Suicide attempts are the most important risk factor for completed suicides^1^; 7%–13% of those who have attempted suicide in the past take their own lives later on.^4^

Trans people, people whose gender identity and/or expression differ from the sex assigned to them at birth, have an elevated suicide risk in comparison to the general population.^5–9^ However, there is a dearth of knowledge about associated factors, particularly concerning protective factors. Suicidality among trans people is associated with high exposure to discrimination, victimization, and violence.^10–12^ A recent large study concluded that family rejection related to gender identity is another important risk for attempted suicide.^13^ Additional risk factors associated with suicidality in the general population, such as depression^14,15^ and substance use,^8^ are thought to be elevated among trans people. The few studies that explored protective factors against suicide among trans people found that social support is important^16–18^ and suicidality decreases with reduced transphobia, with legal gender recognition, and with completion of a needed medical transition.^17^

Data on suicidality among trans people in Sweden are scarce. Existing research reports higher prevalence of completed suicide among transsexual people after gender-affirming surgery compared to cisgender (non-trans) controls.^6^ The majority of studies include individuals with gender dysphoria who pursue medical transition, and rarely include a wider definition of trans people, including gender nonbinary individuals (those who identify with a gender in between male or female, or identify with neither of these genders), people who self-identify as transvestites, and trans people who do not pursue medical gender-affirming interventions. The aim of this study was to investigate the associations between a series of empirically known risk and protective factors and suicidality among trans people in Sweden.

## Methods

### Study setting

In Sweden, adults can change their legal gender to one of two categories, male or female, after undergoing an ∼2-year-long assessment period at one of six gender clinics in the country. To access these clinics, a referral from a psychiatrist is often needed, which is hard to obtain for some people. Due to a large increase in applications, gender clinics have a waiting list of 4–12 months. Since the law was updated in 2013, people are no longer required to undergo sterilization or genital surgery to change their legal gender. When this study was conducted in 2014, people with gender dysphoria who did not fulfill the diagnostic criteria for Transsexualism F64.0 (ICD-10),^19^ identifying as gender nonbinary instead, could not access gender-affirming healthcare. However, this guideline was revised in 2015.^20^ All medical care, including genital and chest surgeries, facial feminization surgery, and fertility preservation, is financed by the national healthcare system. Some important legal developments have occurred in recent years, including a Discrimination Act which protects trans people against discrimination based on gender identity or expression in many sectors, including healthcare.^21^

### Data collection

Participants were self-selected anonymously to a web-based survey between September and November 2014. Participants were recruited via advertisements and email invitations sent by Scandinavia's largest online community for lesbian, gay, bisexual, transgender, and queer (LGBTQ) people (Qruiser). Other LGBTQ organizations promoted the study via different social media channels and personal invitations to email lists. The study was also advertised via Google AdWords and flyers that were distributed at selected gender clinics. The study was designed in close collaboration with experts from the LGBTQ organizations in the country (the Swedish Federation for LGBTQ Rights [RFSL] and the Swedish Youth Federation for LGBTQ rights [RFSL Ungdom]), researchers from Karolinska Institutet, and the Public Health Agency of Sweden. Detailed information on data collection procedures is described elsewhere.^22^

### Study population

Individuals aged 15 and above, who live in Sweden, speak Swedish, and who identified as being or having been trans were eligible to participate in the study. After screening and removing incomplete and noneligible responses, 796 respondents remained for the analysis.^22^

### Ethics

The study protocol, including recruitment and consent procedures, was approved by the Regional Ethical Review Board in Stockholm, Sweden (2014/857-31/5). Participation in the study was made possible after confirming participation by clicking “Yes, I would like to participate in the study” on an electronic consent form. The survey was conducted anonymously and no form of personal identification was collected. The eligible age to participate in the study was 15 years and above, which is the age limit at which no parental or legal guardian consent is required according to Swedish law.

### Dependent variables

Suicidality was defined by suicide ideation during the past 12 months and lifetime suicide attempts. Suicide ideation during the past 12 months was measured by the following question: “Have you during the last 12 months seriously considered taking your own life?” The variable was dichotomized into yes (“yes, once”/“yes, several times”) and no. Lifetime suicide attempts were measured by the following question: “Have you ever tried to take your own life?” The variable was dichotomized into yes (“yes, between the past 2 weeks and 1 year ago”/“yes, more than a year ago”) and no.

### Independent variables

#### Sociodemographic variables

Sociodemographic information was collected, including trans experience, assigned sex at birth, age, county of residence, country of birth (Sweden/other), income, employment status (working/studying, unemployed/long-term sick leave, retired, and other), and education (no high school education, upper-secondary education or some university, and university education ≥3 years). The variable *assigned sex at birth* was collected by asking the respondents about their current legal gender (woman/man) and whether they have changed their legal gender (yes; no, and do not need to; no, but would like to; and no, and cannot change legal gender because the desired gender is not available in Sweden today). Age was collapsed into five categories extracted from participants' reported year of birth. Income was self-reported as net income per month in Swedish Krona, presented here in approximate US dollars. The respondents chose their county of residence from a list of 21 Swedish counties. The variable was later dichotomized into small (<500,000 inhabitants) and large counties (>500,000 inhabitants).

The variable *trans experience* was retrieved from two check-all-that-apply questions regarding respondents' self-reported gender identity and trans experience and was computed into four categories: (1) trans feminine; (2) trans masculine; (3) gender nonbinary; and (4) transvestites. In the Swedish context, the term “transvestite” is a term often used by people who belong to this group themselves. The term refers to individuals who always or periodically express their gender differently from the sex assigned to them at birth. Many, however, do identify with their assigned sex at birth when they are not cross-dressed.

Respondents often checked several boxes for both questions and they had the option to write in a response. The categorization was carried out in a structured way according to specific requirements, which are described in detail elsewhere.^22^

To analyze trans-related victimization, the following variables were included: offensive treatment (exposed to offensive treatment due to gender/trans identity in the past 3 months), fear of discrimination (avoiding different activities due to fear of being discriminated against in the past 12 months), and trans-related violence (lifetime exposure to violence due to trans identity). Time intervals vary as some questions were taken from other national surveys, to make comparisons. Other questions were constructed specifically for this survey. In that case, time intervals were chosen with the intention of capturing respondents' experiences, keeping in mind that recall bias could affect responses.

Stigma was assessed using a modified version of the original 10-item generic stigma consciousness questionnaire for gay men and lesbians.^23^ The questionnaire was shortened and modified by only using four items and by changing the wording from “homophobic” to “transphobic,” from “heterosexual” to “cisgender,” and from “homosexual” to “trans.” Respondents were asked to rate on a seven-grade scale their level of agreement (0 = strongly disagree to 6 = strongly agree) with the following statements: (1) “Stereotypes about trans people have not affected me personally”; (2) “I almost never think about the fact that I am trans when I interact with cisgender people”; (3) “My being trans does not influence how people act toward me”; and (4) “Most cisgender people have a lot more transphobic thoughts than they actually express.” Items 1, 2 and 3 received reverse scores. Higher scores indicated higher levels of stigma consciousness.

#### Trans-related legal and healthcare issues

Access to gender-affirming healthcare was assessed by two questions: (1) “Have you ever sought professional help to get a referral to gender-affirming healthcare?” (“yes”/“no, but I would like to”/“no, I do not need to”) and (2) “Have you ever received a referral to gender-affirming healthcare?” (“yes”/“no”). Respondents who answered “yes” to both questions were categorized as *yes* (having access to gender-affirming healthcare). Respondents who answered “no, but I would like to” to the first question were categorized as *no, but would like to access gender-affirming healthcare* as were respondents who responded “yes” to the first question and “no” to the second question. Those who answered “No, and do not need to” to the first question were categorized as *do not need gender-affirming healthcare*. The section of the questionnaire concerning gender-affirming healthcare was constructed as an extra voluntary section and consequently 116 respondents chose not to complete it. These respondents were categorized as *do not want to answer* and not as missing for the sake of the analysis. Last, the variable *change of legal gender* (“Have you changed your legal gender?”) was also included in the analysis.

#### Other common risk factors

Illicit drug use during the past 6 months was assessed by the question, “Have you, during the last 6 months, used any drugs?” (“no,” “yes,” and “do not want to answer”). In addition, risky consumption of alcohol was measured by the following question: “How many drinks (examples provided) do you have on a typical day when you drink alcohol?” Respondents who reported that they drink more than five drinks were defined as having risky alcohol consumption.

#### Potential protective factors

The generic Life Satisfaction scale, LiSat, was used to measure satisfaction with life as a whole and with nine specific life domains.^24^ Each item was rated on a six-grade ordinal scale ranging from very dissatisfied ( = 0) to very satisfied ( = 6). The variable was dichotomized into satisfied or very satisfied (grades 5–6) versus grades 1–4 (less satisfied). The validity of this dichotomy has been shown previously.^25^ For the items satisfaction with family and satisfaction with partner, missing values were coded together with responses indicating not having a family/partner.

Previous studies found that social support is an important protective factor against suicide among trans individuals.^16–18^ Therefore, information on social support (“Do you have someone you can share your innermost feelings with and confide in?”) and practical support (“Can you get any help from someone if you have a practical problem or are ill? For example, getting advice, borrowing things, help with grocery shopping, repairs, etc.”) was collected. In addition, the variable *social contact with other trans people* was analyzed, as an additional indicator of potentially protective social support. Finally, the variable *openness with trans identity* was analyzed as it has previously been suggested to be associated with psychological wellbeing.^26^

### Data analyses

Stata version 13.1 (StataCorp LLC, College Station, TX) was used for the analysis. Descriptive statistics were carried out to describe the characteristics of the study participants. Univariable logistic regression analysis was performed for the outcomes of suicide ideation in the past 12 months and lifetime suicide attempts against all independent variables. Significant variables (*P* ≤ 0.05) in univariable analysis were later included in a multivariable logistic regression analysis. The measures of association are presented as crude and adjusted odds ratios, with 95% confidence intervals (CIs). A number of covariates were controlled for in the multivariable analysis, regardless of their significance in the univariable analysis. These include age, education, income, employment status, country of birth, risky consumption of alcohol, and illicit drug use during the past 6 months. A Wald's test was used for variables with more than one category in univariable and multivariable analyses, to assess significance of the variable as a whole.

## Results

The sociodemographic characteristics of the study respondents are described in Table 1. Nearly one-fifth (19%) reported ever being exposed to trans-related violence and over half (52%) reported being exposed to offensive treatment during the past 3 months. In addition, fear of discrimination was reported by 64% of respondents. The majority of study respondents reported having social and practical support (72% and 83%, respectively), having social contact with other trans people (77%), and that they are sometimes or always open with their trans identity (51% and 18%, respectively) (Table 2).

**Table 1.** Sociodemographic Characteristics of Study Respondents (*n* = 796)

*Characteristic*	n *(%)*
Trans experience	
Trans feminine	149 (19)
Trans masculine	187 (23)
Gender nonbinary	346 (44)
Transvestite	112 (14)
Missing	2 (0.2)
Assigned sex at birth	
Woman	388 (49)
Man	360 (45)
Missing	48 (6)
Age categories (years)	
15–19	82 (10)
20–29	342 (43)
30–44	202 (25)
45–64	130 (17)
65–94	39 (5)
Missing	1 (0.1)
Country of birth	
Sweden	729 (92)
Other than Sweden	67 (8)
County of residence	
Large county (>500,000 inhabitants)	504 (63)
Small county (<500,000 inhabitants)	269 (34)
Missing	23 (3)
Employment status	
Working/studying	560 (70)
Unemployed/long-term sick leave	156 (20)
Retired	34 (4)
Other	33 (4)
Missing	13 (2)
Education	
No high school education	150 (19)
Upper-secondary education or some university	400 (50)
University education ≥3 years	230 (29)
Other	16 (2)
Monthly net income ($)	
0–1450	425 (53)
1451–2160	98 (12)
2161–3300	145 (18)
>3300	80 (10)
Do not want to answer	44 (6)
Missing	4 (1)

**Table 2.** Distribution of Explanatory Variables Among Study Respondents (*n* = 796)

*Variable*	n *(%)*
Trans-related victimization and stigma	
Offensive treatment past 3 months	
No	363 (46)
Yes	416 (52)
Missing	17 (2)
Fear of discrimination past 12 months	
No	266 (34)
Yes	511 (64)
Missing	19 (2)
Lifetime trans-related violence	
No	613 (77)
Yes	145 (19)
Do not want to answer	19 (2)
Missing	19 (2)
Stigma consciousness (score range 0–24); mean (95% CI)	14.66 (14.27–15.05)
Trans-related legal and healthcare issues	
Access to gender-affirming healthcare	
No, do not need gender-affirming healthcare	129 (16)
Yes	318 (40)
No, but would like to access gender-affirming healthcare	227 (28)
Do not want to answer^a^	116 (15)
Missing	6 (1)
Change of legal gender	
No, and do not need to	157 (20)
Yes	114 (14)
No, but would like to	298 (37)
No, and cannot change legal gender because the desired gender is not available in Sweden today	207 (26)
Do not want to answer	15 (2)
Missing	5 (1)
Other common risk factors	
Illicit drug use past 6 months	
No	696 (87)
Yes	65 (8)
Do not want to answer	12 (2)
Missing	23 (3)
Risky alcohol consumption	
No	630 (79)
Yes	153 (19)
Missing	13 (2)
Potential protective factors	
Life Satisfaction scale (LiSat)	
Life in general	
Satisfied	240 (30)
Less satisfied	536 (67)
Missing	20 (3)
Occupation	
Satisfied	285 (36)
Less satisfied	488 (61)
Missing	23 (3)
Finances	
Satisfied	186 (23)
Less satisfied	590 (74)
Missing	20 (3)
Recreational time	
Satisfied	233 (29)
Less satisfied	544 (68)
Missing	19 (2)
Contact with friends and acquaintances	
Satisfied	307 (39)
Less satisfied	470 (59)
Missing	19 (2)
Sex life	
Satisfied	181 (23)
Less satisfied	593 (74)
Missing	22 (3)
Physical health	
Satisfied	186 (23)
Less satisfied	591 (74)
Missing	19 (2)
Psychological wellbeing	
Satisfied	197 (25)
Less satisfied	580 (73)
Missing	19 (2)
Family life	
Satisfied	253 (32)
Less satisfied	381 (48)
Have no family/missing^b^	162 (20)
Relationship with partner	
Satisfied	269 (34)
Less satisfied	226 (28)
Have no partner/missing^c^	301 (38)
Practical support	
Always	320 (40)
Most of the time	341 (43)
Almost never	87 (11)
Never	28 (4)
Missing	20 (2)
Social support	
Yes	572 (72)
No	205 (26)
Missing	19 (2)
Social contact with other trans people	
Yes	614 (77)
No	166 (21)
Missing	16 (2)
Open with trans identity	
Always open	140 (18)
Sometimes open	406 (51)
Rarely open	137 (17)
Never open	51 (6)
Trans identity shows	37 (5)
Missing	25 (3)

^a^ In the questionnaire, the section concerning gender-affirming healthcare was an additional voluntary section. Respondents who chose not to complete it were coded as do not want to answer.

^b^ Missing values in family satisfaction were combined with responses indicating not having a family.

^c^ Missing values in partner satisfaction were combined with responses indicating not having a partner.

CI, confidence interval.

The majority of respondents (67%) reported less satisfaction with life in general. In addition, 74% reported less satisfaction with their finances, 61% with their occupation, and 68% with their recreational activities. Over half (59%) reported less satisfaction with their contacts with friends and acquaintances, 74% with their sex life, 48% with their family life, and 28% with their relationship with their partner. Last, 74% reported less satisfaction with their physical health and 73% with their psychological wellbeing (Table 2).

### Suicide ideation in the past 12 months

A total of 37% of respondents reported having seriously considered suicide in the past year. Crude and adjusted analyses of associated factors are presented in Table 3. Multivariable regression analysis showed that unemployment or long-term sick leave (adjusted odds ratio [aOR] = 2.62; 95% CI: 1.53–4.50), country of birth other than Sweden (aOR = 2.27; 95% CI: 1.09–4.74), and risky alcohol consumption (aOR = 2.16; 95% CI: 1.30–3.60) were significantly associated with suicide ideation. Older age was significantly associated with decreased risk of suicide ideation (aOR = 0.11; 95% CI: 0.04–0.30).

**Table 3.** Univariable and Multivariable Associations with Suicide Ideation in the Past Twelve Months and Lifetime Suicide Attempts

*Variable*	*Suicide ideation past 12 months Yes* n *(%)*^a^	*Crude OR (95% CI)*	*Adjusted OR (95% CI)*	*Lifetime suicide attempts Yes* n *(%)*^a^	*Crude OR (95% CI)*	*Adjusted OR (95% CI)*
Country of birth						
Sweden	256 (36)	1.00 (REF)		231 (32)	1.00 (REF)	
Other	28 (44)	1.38 (0.82–2.31)	2.27^*^ (1.09–4.74)	21 (32)	0.97 (0.57–1.67)	1.15 (0.58–2.28)
Age groups						
15–19	46 (57)	1.00 (REF)		32 (40)	1.00 (REF)	
20–29	139 (41)	0.53 (0.33–0.87)	0.54 (0.26–1.12)	126 (37)	0.91 (0.55–1.50)	1.06 (0.53–2.10)
30–44	68 (35)	0.40 (0.24–0.69)	0.47 (0.20–1.12)	62 (31)	0.69 (0.40–1.18)	1.28 (0.58–2.85)
45–64	27 (22)	0.21 (0.12–0.40)	0.11^***^ (0.04–0.30)	26 (21)	0.41 (0.22–0.76)	0.60 (0.24–1.51)
65–94	4 (11)	0.10 (0.03–0.30)	0.04^*^ (0.002–0.75)	6 (17)	0.31 (0.11–0.82)	1.80 (0.18–17.74)
Employment status						
Working/studying	173 (32)	1.00 (REF)		159 (29)	1.00 (REF)	
Unemployed/long-term sick leave	88 (58)	3.03 (2.09–4.38)	2.62^***^ (1.53–4.50)	75 (50)	2.43 (1.68–3.52)	2.52^***^ (1.53–4.15)
Retired	4 (13)	0.33 (0.11–0.97)	3.36 (0.21–52.43)	5 (16)	0.47 (0.18–1.26)	0.34 (0.30–3.87)
Other	13 (41)	1.48 (0.72–3.07)	0.91 (0.33–2.51)	9 (27)	0.92 (0.42–2.03)	0.53 (0.20–1.39)
Monthly net income ($)						
0–1450	192 (46)	1.72 (1.08–2.74)	0.90 (0.48–1.69)	159 (38)	1.36 (0.85–2.19)	1.05 (0.58–1.90)
1451–2160	32 (33)	1.00 (REF)		30 (31)	1.00 (REF)	
2161–3300	27 (19)	0.48 (0.26–0.88)	0.48 (0.21–1.09)	33 (24)	0.70 (0.39–1.24)	1.03 (0.49–2.16)
>3300	15 (20)	0.49 (0.24–1.00)	1.96 (0.73–5.27)	12 (16)	0.41 (0.19–0.87)	1.27 (0.50–3.27)
Do not want to answer	15 (34)	1.03 (0.49–2.20)	0.61 (0.19–1.90)	15 (35)	1.20 (0.56–2.56)	2.13 (0.77–5.88)
Education						
No high school education	67 (46)	1.49 (1.01–2.19)	1.80 (0.98–3.29)	56 (38)	1.19 (0.80–1.76)	1.40 (0.82–2.39)
Upper-secondary education or some university	140 (36)	1.00 (REF)		132 (34)	1.00 (REF)	
University education ≥3 years	71 (32)	0.84 (0.59–1.19)	1.44 (0.86–2.40)	58 (26)	0.68 (0.47–0.98)	0.77 (0.48–1.26)
Other	6 (38)	1.07 (0.38–3.00)	0.93 (0.21–4.05)	6 (38)	1.17 (0.42–3.30)	1.32 (0.38–4.57)
Illicit drug use past 6 months						
No	236 (34)	1.00 (REF)		212 (31)	1.00 (REF)	
Yes	40 (63)	3.20 (1.88–5.43)	1.96 (0.99–3.88)	34 (52)	2.49 (1.49–4.15)	2.31^*^ (1.21–4.39)
Do not want to answer	6 (50)	1.91 (0.61–6.01)	0.98 (0.21–4.51)	6 (50)	2.27 (0.72–7.11)	1.13 (0.26–4.79)
Risky alcohol consumption						
No	201 (33)	1.00 (REF)		180 (29)	1.00 (REF)	
Yes	76 (51)	2.09 (1.46–3.00)	2.16^**^ (1.30–3.60)	66 (43)	1.82 (1.27–2.62)	1.76^*^ (1.11–2.80)
Trans experience						
Trans feminine	60 (41)	1.00 (REF)		54 (37)	1.00 (REF)	
Trans masculine	75 (41)	0.98 (0.63–1.52)		79 (42)	1.26 (0.80–1.96)	
Gender nonbinary	129 (38)	0.88 (0.59–1.30)		105 (31)	0.76 (0.50–1.14)	
Transvestite	19 (19)	0.33 (0.18–0.60)		13 (13)	0.24 (0.12–0.48)	
Offensive treatment past 3 months						
No	88 (25)	1.00 (REF)		87 (24)	1.00 (REF)	
Yes	195 (47)	2.72 (2.00–3.70)	1.77^**^ (1.10–2.85)	164 (40)	2.06 (1.51–2.81)	1.58^*^ (1.02–2.46)
Lifetime trans-related violence						
No	196 (32)	1.00 (REF)		172 (28)	1.00 (REF)	
Yes	79 (55)	2.59 (1.79–3.75)	1.99^*^ (1.17–3.40)	68 (47)	2.24 (1.55–3.25)	1.77^*^ (1.09–2.86)
Do not want to answer	9 (47)	1.89 (0.75–4.71)	2.76 (0.73–10.42)	11 (58)	3.50 (1.38–8.85)	4.20^*^ (1.18–14.90)
Fear of discrimination						
No	61 (23)	1.00 (REF)		64 (24)	1.00 (REF)	
Yes	220 (43)	2.52 (1.80–3.53)		186 (36)	1.79 (1.28–2.51)	
Stigma consciousness		1.08 (1.05–1.11)			1.06 (1.03–1.09)	
Access to gender-affirming healthcare						
No, do not need gender-affirming healthcare	32 (25)	1.00 (REF)		23 (18)	1.00 (REF)	
Yes	127 (41)	2.02 (1.27–3.20)		129 (41)	3.12 (1.89–5.17)	1.96 (0.90–4.24)
No, but would like to access gender-affirming healthcare	87 (40)	1.95 (1.20–3.17)		65 (29)	1.86 (1.09–3.19)	1.51 (0.75–3.06)
Do not want to answer^b^	34 (30)	1.28 (0.72–2.26)		32 (29)	1.79 (0.97–3.30)	2.35^*^ (1.10–5.00)
Change of legal gender						
No, and do not need to	30 (20)	1.00 (REF)		23 (15)	1.00 (REF)	
Yes	34 (30)	1.71 (0.97–3.01)		43 (38)	3.45 (1.92–6.18)	
No, but would like to	139 (48)	3.65 (2.30–5.80)		116 (40)	3.70 (2.24–6.10)	
No, and cannot change legal gender because the desired gender is not available in Sweden today	77 (38)	2.42 (1.48–3.96)		64 (32)	2.60 (1.52–4.43)	
Do not want to answer	3 (21)	1.08 (0.28–4.12)		4 (27)	2.04 (0.60–6.95)	
Life Satisfaction scale (LiSat)						
Life in general						
Satisfied	31 (13)	1.00 (REF)		59 (25)	1.00 (REF)	
Less satisfied	250 (47)	5.96 (3.94–9.02)		190 (36)	1.68 (1.19–2.37)	
Occupation						
Satisfied	69 (24)	1.00 (REF)		81 (29)	1.00 (REF)	
Less satisfied	212 (44)	2.43 (1.75–3.36)		168 (35)	1.32 (0.96–1.82)	
Finances						
Satisfied	42 (23)	1.00 (REF)		42 (23)	1.00 (REF)	
Less satisfied	240 (41)	2.31 (1.58–3.39)		208 (35)	1.86 (1.27–2.73)	
Recreational time						
Satisfied	48 (21)	1.00 (REF)		61 (26)	1.00 (REF)	
Less satisfied	234 (43)	2.91 (2.03–4.17)		189 (35)	1.50 (1.06–2.11)	
Contact with friends and acquaintances						
Satisfied	74 (25)	1.00 (REF)		83 (27)	1.00 (REF)	
Less satisfied	208 (44)	2.45 (1.78–3.38)	1.71^*^ (1.05–2.78)	167 (36)	1.50 (1.09–2.06)	
Sex life						
Satisfied	43 (24)	1.00 (REF)		53 (29)	1.00 (REF)	
Less satisfied	238 (41)	2.18 (1.49–3.19)		195(33)	1.19 (0.83–1.71)	
Psychological wellbeing						
Satisfied	17 (9)	1.00 (REF)		35 (18)	1.00 (REF)	
Less satisfied	265 (46)	8.98 (5.32–15.16)	3.60^***^ (1.70–7.64)	214 (37)	2.70 (1.80–4.04)	
Physical health						
Satisfied	33 (18)	1.00 (REF)		36 (19)	1.00 (REF)	
Less satisfied	249 (42)	3.35 (2.22–5.06)		213 (36)	2.37 (1.58–3.53)	
Family						
Satisfied	59 (24)	1.00 (REF)		64 (25)	1.00 (REF)	
Less satisfied	175 (46)	2.81 (1.98–4.02)		150 (40)	1.92 (1.36–2.73)	
Have no family/missing^c^	50 (34)	1.69 (1.08–2.66)		38 (26)	1.01 (0.64–1.61)	
Partner						
Satisfied	68 (26)	1.00 (REF)		75 (28)	1.00 (REF)	
Less satisfied	93 (42)	2.08 (1.42–3.05)		80 (36)	1.45 (0.99–2.12)	
Have no partner/missing^d^	123 (43)	2.21 (1.54–3.17)		97 (34)	1.32 (0.92–1-90)	
Practical support						
Always	86 (27)	1.00 (REF)		91 (29)	1.00 (REF)	
Most of the time	133 (39)	1.74 (1.25–2.41)		103 (30)	1.09 (0.78–1.52)	0.80 (0.53–1.23)
Almost never	48 (56)	3.38 (2.06–5.53)		38 (44)	1.94 (1.19–3.17)	1.46 (0.76–2.78)
Never	14 (50)	2.67 (1.22–5.84)		16 (57)	3.34 (1.52–7.34)	4.03^**^ (1.45–11.16)
Social support						
Yes	194 (34)	1.00 (REF)		177 (31)	1.00 (REF)	
No	87 (43)	1.47 (1.06–2.04)		71 (35)	1.17 (0.84–1.64)	
Open with trans identity						
Always open	54 (39)	1.00 (REF)		53 (38)	1.00 (REF)	
Sometimes open	148 (37)	0.92 (0.62–1.36)		127 (31)	0.75 (0.50–1.12)	
Rarely open	42 (31)	0.70 (0.43–1.16)		42 (31)	0.74 (0.45–1.22)	
Never open	14 (29)	0.63 (0.31–1.28)		10 (20)	0.40 (0.18–0.86)	
Trans identity shows	23 (62)	2.58 (1.22–5.45)		17 (46)	1.39 (0.67–2.90)	
Social contact with other trans people						
Yes	221 (36)	1.00 (REF)		192 (31)	1.00 (REF)	
No	62 (38)	1.08 (0.75–1.54)		59 (36)	1.21 (0.85–1.75)	

^a^ % Presented as column percentages.

^b^ In the questionnaire, the section concerning gender-affirming healthcare was an additional voluntary section. Respondents who chose not to complete it were coded as do not want to answer.

^c^ Missing values in family satisfaction were combined with responses indicating not having a family.

^d^ Missing values in partner satisfaction were combined with responses indicating not having a partner.

^*^ *P* < 0.05, ^**^P < 0.01, ^***^P < 0.001.

OR, odds ratio.

After controlling for the above-mentioned covariates, the following variables remained significant to suicide ideation in the past 12 months: offensive treatment in the past 3 months (aOR = 1.77; 95% CI: 1.10–2.85), lifetime exposure to trans-related violence (aOR = 1.99; 95% CI: 1.17–3.40), less satisfaction with contacts with friends and acquaintances (aOR = 1.71; 95% CI: 1.05–2.78), and less satisfaction with own psychological wellbeing (aOR = 3.60; 95% CI: 1.70–7.64).

### Lifetime suicide attempts

Nearly a third (32%) of the respondents reported ever attempting a suicide (Table 3). Unemployment or long-term sick leave (aOR = 2.52; 95% CI: 1.53–4.15), illicit drug use in the past 6 months (aOR = 2.31; 95% CI: 1.21–4.39), and risky alcohol consumption (aOR = 1.76; 95% CI: 1.11–2.80) were significantly associated with lifetime suicide attempts. The following variables remained significantly associated with lifetime suicide attempts, independent of the above-mentioned covariates: offensive treatment in the past 3 months (aOR = 1.58; 95% CI: 1.02–2.46), lifetime exposure to trans-related violence (aOR = 1.77; 95% CI: 1.09–2.86), and never having practical support (aOR = 4.03; 95% CI: 1.45–11.16). The modified stigma-consciousness scale did not maintain significance in multivariable analysis for the outcomes of suicide ideation and suicide attempts.

## Discussion

We found that trans respondents included in our study face health inequities, including severe consequences such as increased suicidality. Similar to findings from other settings,^11,17,27,28^ our results show that suicidality among trans people is disproportionately high. When compared to the general Swedish population,^3^ the prevalence of suicide ideation in the past year is nearly seven and a half fold higher among trans respondents. Our findings demonstrate that trans-related offensive treatment and violence are significant correlates of suicidality. These findings correspond with Clements-Nolle et al.'s findings that verbal and physical gender victimization are associated with suicide attempts.^27^ Similarly, Maguen and Shipherd found that trans-related violence was associated with past suicide attempts,^11^ while Bauer et al. found that reduced transphobia resulted in reduced suicidality.^17^ These results emphasize the important mechanisms by which targeted victimization causes poor mental health and increased suicidality among sexual and gender minorities, as explained by Meyer's minority stress theory.^29^

Furthermore, the finding that trans-related victimization in a Swedish trans population, irrespective of access to gender-affirming healthcare, is associated with suicidality adds important information to previous Swedish findings. Dhejne et al. found that suicide attempts and mortality caused by suicide and psychiatric morbidity, adjusted for immigrant status and prior psychiatric morbidity, were higher among transsexual patients after gender-affirming surgery compared to matched, unexposed cisgender controls.^6^ It is important to point out that the analysis in Dhejne et al.'s^6^ study was not adjusted for history of victimization. These findings have been misinterpreted and used to deny trans people gender-affirming surgery. It is likely that the population in this study and in Dhejne et al.'s study is partly overlapping and a cautious interpretation would be that trans-related victimization could explain some of the increased vulnerability to suicide attempts and completed suicide that was found in Dhejne et al.'s study.^6^ It is also of importance to highlight that psychiatric morbidity is more prevalent among people applying for gender-affirming medical interventions.^14^ While some psychiatric morbidity could be explained by gender dysphoria caused by gender incongruence per se, some are related to other challenges, which if not addressed adequately could remain even after medical transition.

To our knowledge, this is the first study to analyze life satisfaction in trans people using the LiSat. Even though different methodologies were applied, study respondents reported less satisfaction with life in general and in various life domains compared to the general Swedish population.^25^ Our findings show that less satisfaction with contacts with friends and acquaintances is associated with past-year suicide ideation. Moreover, never having practical support was associated with elevated suicide attempts. These findings suggest that a social network that offers both emotional and practical support is important and they provide additional evidence that social support is a protective factor against suicidality.^16–18^ Less satisfaction with psychological wellbeing was also associated with past-year suicide ideation. Depression and anxiety, both associated with suicidality,^27^ are common conditions among trans people.^14^

Past-year suicide ideation correlated with age, with older respondents having significantly decreased risk compared to younger respondents. Other studies found increased suicidality among Swedish youth^30^ and among sexual minority youth specifically.^31^ This finding emphasizes the importance of adapted, targeted interventions for young trans people, provision of support for families with trans children, and actions to eliminate violence and discrimination in schools to overcome this severe mental health issue.

Suicide ideation was significantly more common among respondents born outside of Sweden. It has previously been shown that immigrants in Europe have an elevated risk of psychiatric disorders as well as higher rates of suicide attempts compared to their hosts in the country of residence.^32,33^ These elevated vulnerabilities are largely explained by psychological distress related in some way to the migration process and to adverse social circumstances.^32,33^ In the case of our study, foreign-born respondents might face multiple types of challenges related to their trans identities and foreign background, which in turn may result in increased vulnerability to suicidality.

We found no significant difference in suicidality between groups with different trans experiences or juridical statuses. Similarly, previous studies found no difference in psychiatric disorders and psychopathology between trans-masculine and trans-feminine individuals.^14^ This suggests that trans people are equally vulnerable and interventions must target all trans people.

A series of hypothesized contributors were found to be insignificant in the multivariable analysis; legal gender recognition and access to gender-affirming healthcare, surprisingly, were insignificant to suicidality. This finding contradicts Bauer et al.'s findings that having any personal identification documents changed to the appropriate sex and completing a medical transition are associated with reduced suicide risk.^17^ It is important to underline that we did not measure completion of medical transition. Instead, we analyzed whether one has the desire to and has started the bureaucratic procedure of seeking gender-affirming healthcare. This could, perhaps, partially explain the lack of association. Other studies report improved mental health for trans people with gender dysphoria who have transitioned medically.^14,34,35^

Last, we attempted to assess stigma consciousness using a modified version of the stigma consciousness questionnaire for gay men and lesbians and found no significant associations between stigma consciousness and suicidality. The modified scale had never been validated and perhaps did not truly capture the stigma experienced by respondents. Future studies should validate trans-specific stigma assessment tools to investigate further the complex mechanisms by which stigma may cause ill health.^36^

### Strengths and limitations

This study has both strengths and limitations. Despite utilizing numerous methods of recruitment and reaching a diverse group of respondents, this study employed convenience sampling and, therefore, we cannot draw any conclusions about the representativeness of the study sample to the larger Swedish trans population. Trans people not born in Sweden were represented to a smaller extent, suggesting that the availability of the questionnaire in Swedish alone might have compromised participation in this group. Another limitation is related to the temporality of the analyzed variables. The sequence of the studied events is unknown; it is possible that suicide attempts in the past 12 months occurred before being exposed to violence. The results should, therefore, be interpreted with caution, as should other results retrieved from cross-sectional data.

Our study did not include data regarding psychiatric morbidity, which is an important risk factor for suicidality. However, satisfaction with psychological wellbeing was included, which could be seen as an indicator of the absence of psychiatric morbidity. In addition, risky alcohol consumption and illicit drug use, which are other known confounders, were analyzed.

Our study also presents major strengths. This is, by far, the largest survey targeting trans people in Sweden. The design of this study is unique in that it was carried out in close collaboration with experts from LGBTQ organizations, the Public Health Agency of Sweden, and researchers within academia. This collaboration enhances the quality and credibility of this study and ensures the dissemination of the results to decision-makers and organizations who cater to trans communities.

## Conclusions

The prevalence of suicide ideation and attempts among trans respondents is disproportionately high and is directly correlated with trans-related victimization. Preventing targeted victimization is, therefore, a key preventive intervention. Our findings illuminate that it is crucial to reverse health inequities and promote prevention of suicide by societal engagement in destigmatizing activities and promoting trans-sensitive healthcare, suicide prevention interventions, and mental health services. This priority is recognized by the Swedish government; suicide prevention is coordinated on a national level by the Public Health Agency, which is mandated to analyze health and health determinants routinely among LGBTQ persons. In addition, the Public Health Agency of Sweden is responsible for identifying specific preventive strategies against suicide and suicide attempts among trans people. Our findings of associated risk factors for suicidality are important; however, finding protective factors against suicidality is equally important and research focusing on that is scarce. We, therefore, encourage quantitative and qualitative research focusing on protective factors, to tailor specific and appropriate interventions against elevated suicidality among trans people.
